# Silent Post-intubation Tracheal Stenosis Unmasked by Status Epilepticus in a Paediatric Neurotrauma Survivor: A Triple Jeopardy Airway Crisis

**DOI:** 10.7759/cureus.111691

**Published:** 2026-06-28

**Authors:** Anindya Dasgupta, Bodhisatwa Choudhuri, Swarup Paul, Abhradip Das, Pranesh Sarkar, Prasun Banerjee, Biswarup Boxi, Sagnik Datta, Siddhartha Basu

**Affiliations:** 1 Emergency Medicine, Institute of Neurosciences Kolkata, Kolkata, IND; 2 Critical Care, Emergency Medicine and Rheumatology, Parkview Super Speciality Hospital, Kolkata, IND; 3 Critical Care Medicine, Narayana Multispeciality Hospital, Barasat, IND; 4 Pulmonology, Narayana Multispeciality Hospital, Barasat, IND; 5 Emergency Medicine, Narayana Multispeciality Hospital, Barasat, IND; 6 Emergency Medicine, Woodlands Hospital, Kolkata, IND

**Keywords:** bronchoscopy, cannot intubate cannot oxygenate, difficult airway, emergency tracheostomy, paediatric airway, post-intubation tracheal stenosis, respiratory acidosis, status epilepticus, tracheal stenosis, traumatic brain injury

## Abstract

Post-intubation tracheal stenosis (PITS) can remain clinically silent until a physiological stressor precipitates life-threatening airway failure. This case report describes a 15-year-old male, a survivor of traumatic brain injury requiring prolonged endotracheal intubation, who presented in active convulsive status epilepticus with a Glasgow Coma Scale score of 4 and oxygen saturation of 43%. Sequential endotracheal intubation with three tube sizes failed, establishing a cannot intubate, cannot oxygenate (CICO) scenario. Emergency flexible bronchoscopy, performed with a laryngeal mask airway as a ventilatory bridge, revealed fully mobile vocal cords but a pinpoint subglottic lumen through which the bronchoscope could not advance, consistent with Myer-Cotton Grade III-IV PITS. Critical respiratory acidosis was confirmed: pH 6.95, partial pressure of carbon dioxide (pCO_2_) 135 mmHg. Emergency surgical tracheostomy under local anaesthesia restored ventilation. CT of the neck confirmed circumferential tracheal wall thickening extending 2.3 cm below the subglottis. The patient was weaned from mechanical ventilation and discharged on hospital day 11 with planned outpatient tracheal reconstruction. This case introduces the triple jeopardy airway crisis: the convergence of active status epilepticus, occult mechanical airway obstruction, and critical respiratory acidosis, and illustrates that successful CICO management in this setting demands anatomical contextualisation beyond algorithmic adherence.

## Introduction

Post-intubation tracheal stenosis (PITS) is a recognised complication of prolonged endotracheal intubation, with a reported incidence of 10% to 22% in non-COVID-19 patients and rising further with increased mechanical ventilation requirements [1,2]. The underlying mechanism involves cuff-induced mucosal ischaemia followed by tracheal inflammation and progressive fibrotic luminal narrowing [3]. A defining clinical feature is its silent natural history: stenosis typically becomes symptomatic only when luminal diameter falls below approximately 5 to 6 mm, at which point physiological reserve is precipitously exhausted [3]. In the paediatric population, this diagnostic silence is further compounded by misattribution of progressive dyspnoea and stridor to post-viral reactive airway disease or asthma [4].

When PITS converges with an acute neurological emergency such as status epilepticus (SE), defined as continuous or recurrent seizure activity lasting more than five minutes without recovery of consciousness [5], the result is a cannot intubate, cannot oxygenate (CICO) crisis requiring immediate front-of-neck airway (FONA) access [6]. In the paediatric patient, smaller laryngotracheal dimensions impose additional technical challenge [7].

The simultaneous occurrence of active SE, severe mechanical airway obstruction from unrecognised PITS, and critical respiratory acidosis constitutes what the authors propose as the triple jeopardy airway crisis - a descriptive conceptual framework arising from this single case observation, which requires prospective evaluation in larger cohorts before generalised application: a construct not previously described in a paediatric neurotrauma survivor. Written informed consent for publication was obtained from the patient’s legal guardian.

## Case presentation

A 15-year-old male patient with a background of traumatic brain injury (TBI) sustained in a road traffic accident presented as a walk-in to the emergency department in active convulsive status epilepticus with gasping respirations. The index TBI had required left frontal craniotomy for evacuation of an extradural haematoma with pneumocephalus six months ago; he had been maintained on levetiracetam following neurosurgical discharge. He had been progressively drowsy at a peripheral facility in the days preceding presentation, suggesting a sub-acute onset prior to acute decompensation.

On arrival, the Glasgow Coma Scale (GCS) score was 4 (E1V1M2) and peripheral oxygen saturation (SpO_2_) was 43% on room air, with markedly reduced bilateral air entry. Arterial blood gas (ABG) analysis demonstrated critical respiratory acidosis: pH 6.95, partial pressure of carbon dioxide (pCO_2_) 135 mmHg (Table 1).

**Table 1:** Key investigations for a 15-year-old male presenting with status epilepticus and unrecognised severe subglottic tracheal stenosis. BE, base excess; CBC, complete blood count; CRP, C-reactive protein; HCO_3_−, bicarbonate; K, potassium; Na, sodium; pCO_2_, partial pressure of carbon dioxide; pO_2_, partial pressure of oxygen; WBC, white blood cell. ↑ above reference range; ↓ below reference range.

Investigation	Parameter	On presentation (day 1)	Hospital day 3	Hospital day 8	Reference range
Arterial blood gas	pH	6.95 ↓	7.26 ↓	7.41	7.35-7.45
	pCO_2_ (mmHg)	135 ↑	47	36	35-45
	pO_2_ (mmHg)	153	408 ↑	105	80-100 (room air)
	HCO_3_- (mmol/L)	28.8	20.4	22.1	22-26
	BE (mmol/L)	−3.3 ↓	-1.4	0.6	−2 to +2
	Lactate (mmol/L)	2.6 ↑	1.6	1.1	<2.0
Haematology (CBC)	Haemoglobin (g/dL)	10.2 ↓	10.6 ↓	13.3	13.0-17.0
	WBC (×10^9^/L)	11.4	9.3	5.7	4.0-11.0
	Neutrophils (%)	81 ↑	68	45.1	50-70
	Eosinophils (%)	2.1	1.2	7.5 ↑	1-4
	Platelets (×10^9^/L)	187	170	252	150-400
Biochemistry	Serum Na (mmol/L)	142	139	141	135-145
	Serum K (mmol/L)	4.38	2.8 ↓	4.7	3.5-5.0
	Serum creatinine (mg/dL)	0.63	0.72	0.64	0.6-1.2
	Serum total bilirubin (mg/dL)	1.1	0.8	0.7	0.3-1
	CRP (mg/L)	11.9 ↑	10.2 ↑	7.0 ↑	<5.0

**Figure 1:**
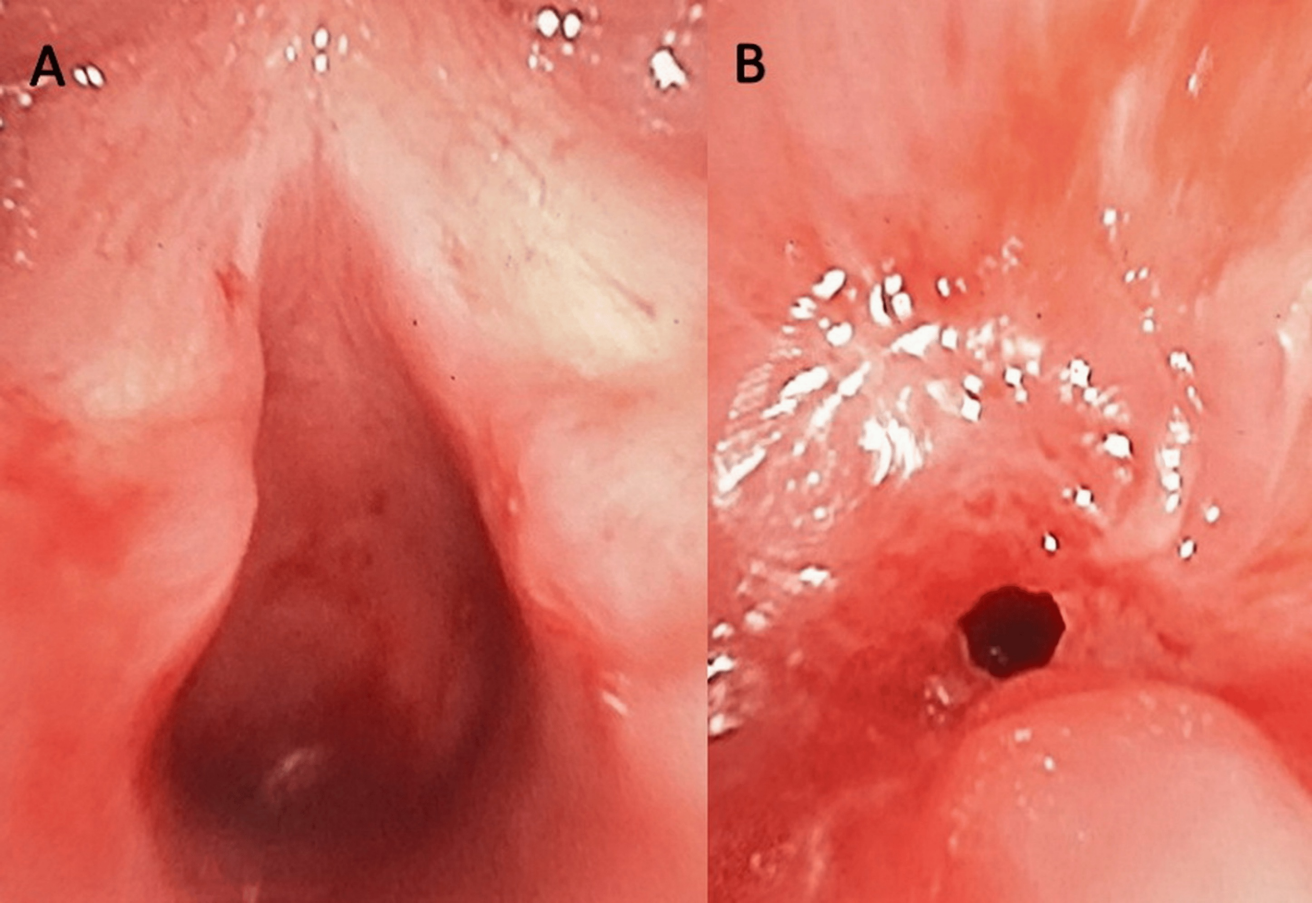
Bronchoscopic findings of post-intubation subglottic tracheal stenosis in a 15-year-old male. (A) Mobile vocal cords visualised through the laryngeal mask airway, confirming that the level of obstruction was subglottic rather than glottic. (B) Pinpoint subglottic tracheal lumen with near-total luminal obliteration, through which the bronchoscope could not be advanced, consistent with at minimum Myer-Cotton Grade III post-intubation tracheal stenosis.

Sequential endotracheal intubation with three tube sizes failed (sizes 8.0, 7.5, and 7.0), establishing a CICO scenario. A laryngeal mask airway (LMA) was inserted as a bridge. Emergency bedside flexible bronchoscopy revealed fully mobile vocal cords (Figure 1A) but a pinpoint subglottic tracheal lumen through which the bronchoscope could not be advanced (Figure 1B). Emergency surgical tracheostomy was performed under local anaesthesia; placement was confirmed by repeat bronchoscopy with direct visualisation of the carina. 

The patient was admitted to the intensive care unit (ICU) on mechanical ventilation. Computed tomography (CT) of the neck with contrast, performed on hospital day 2, confirmed circumferential heterogeneously enhancing wall thickening of the upper trachea extending 2.3 cm below the subglottis, consistent with PITS. CT of the chest demonstrated bilateral lower lobe ground-glass opacities (GGOs) consistent with aspiration pneumonitis. Full investigations are detailed in Table 1; the ICU management timeline is detailed in Table 2.

**Table 2:** Intensive care unit management timeline for a 15-year-old male presenting with status epilepticus and unrecognised severe subglottic tracheal stenosis. ABG, arterial blood gas; CICO, cannot intubate, cannot oxygenate; CPAP, continuous positive airway pressure; CT, computed tomography; CTVS, cardiothoracic and vascular surgery; ENT, ear, nose, and throat; ET, endotracheal; FiO_2_, fraction of inspired oxygen; GGO, ground-glass opacity; ICU, intensive care unit; IV, intravenous; K, potassium; KCl, potassium chloride; LMA, laryngeal mask airway; Na, sodium; NGT, nasogastric tube; pCO_2_, partial pressure of carbon dioxide; PITS, post-intubation tracheal stenosis; SpO_2_, peripheral oxygen saturation; V-A/C, volume-controlled assist-control; WBC, white blood cell.

Hospital day	Clinical phase	Key medications	Key clinical events
Day 1	Emergency resuscitation	IV Levetiracetam 2 g (load); IV Lorazepam 4 mg; IV Midazolam 3 mg; IV Atracurium 50 mg; IV Fentanyl 100 mcg; IV Piperacillin-Tazobactam 4.5 g; IV Paracetamol 1 g; IV Pantoprazole 40 mg	CICO declared; LMA bridge inserted; emergency flexible bronchoscopy — pinpoint subglottic stenosis confirmed; emergency surgical tracheostomy under local anaesthesia; V-A/C ventilation commenced (Rate 18; FiO_2_ 50%); ICU admission; NGT + urinary catheter; family counselled (very guarded prognosis)
Day 2	Emergency resuscitation	Piperacillin-Tazobactam continued; Atracurium + Fentanyl infusions; IV KCl supplementation	CT Brain, CT Neck (contrast), CT Chest performed; hypokalaemia (K 2.8 mmol/L) identified and corrected; ABG markedly improved on mechanical ventilation
Days 3-5	Pulmonary stabilisation	Antibiotics and sedation infusions continued; ventilator rate reduced (Day 4)	ET secretion and urine cultures — no growth at 48 h; Day 4: respiratory alkalosis identified (pH 7.53; pCO_2_ 26 mmHg) and corrected by ventilator adjustment; Day 5: ABG normalised; sedation weaning commenced
Days 6-7	Weaning and recovery	Sedation weaned and ceased	Transitioned to CPAP mode; neurological status improving; chest and limb physiotherapy commenced
Days 8-9	Weaning and recovery	Topical antifungal commenced	T-piece trial tolerated; SpO_2_ ≥98% sustained; peripheral eosinophilia (7.5%) identified — flagged for outpatient haematological review; cutaneous fungal infection noted on abdomen
Day 10-11	Step-down and discharge	Levetiracetam resumed orally; physiotherapy continued	Transferred to high-dependency unit; tracheostomy in situ; ENT and CTVS multidisciplinary review; definitive tracheal resection with end-to-end anastomosis planned as outpatient; family counselled on long-term prognosis; discharged in stable condition

## Discussion

Aetiology of the subglottic stenosis

PITS was the primary aetiological diagnosis, supported by the documented history of prolonged endotracheal intubation, the subglottic location of the lesion at the endotracheal tube cuff site, and the circumferential CT morphology: classical features of cuff-induced fibrotic narrowing [8]. Post-tracheostomy stenosis was excluded, as the patient had no prior tracheostomy. Idiopathic subglottic stenosis was considered unlikely, as it predominantly affects middle-aged women without prior intubation history [9]. Inflammatory aetiologies, including granulomatosis with polyangiitis and sarcoidosis, were excluded on the basis of absent systemic features, a normal baseline inflammatory profile, and the anatomically precise cuff-site location. Malignant causes were excluded by the patient’s age and CT appearances of smooth circumferential thickening without luminal irregularity or mediastinal lymphadenopathy [8].

Cause of seizure activity and respiratory failure

Respiratory failure resulted from PITS acting as a fixed mechanical upper airway obstruction, causing progressive hypoventilation and CO_2_ retention as ventilatory demand exceeded the capacity of the critically narrowed airway. The acute seizure presentation was most likely precipitated by hypoxic-hypercapnic encephalopathy: at presentation, SpO_2_ was 43% and pCO_2_ 135 mmHg, derangements sufficient to independently cause loss of consciousness and convulsive activity. The patient's background of TBI and prior craniotomy conferred an established predisposition to post-traumatic epilepsy, occurring in up to 20% of patients [10]. Both mechanisms may have contributed simultaneously: post-traumatic epilepsy as a permissive substrate that lowered seizure threshold, and hypoxic-hypercapnic encephalopathy as the probable acute precipitant, given the severity of the physiological derangements at presentation. The absence of recurrent seizures following airway rescue and oxygenation restoration supports the latter as the dominant proximate trigger. Secondary intracranial haemorrhage was excluded by CT brain, demonstrating no new haemorrhage or mass effect. Hyponatraemia was excluded by a normal serum sodium at presentation.

Aetiology of the pulmonary infiltrates

Bilateral lower lobe GGOs in the context of prolonged ictal state, loss of airway protective reflexes, and supine positioning were consistent with aspiration pneumonitis rather than established bacterial pneumonia [11]. Sterile endotracheal secretion cultures at 48 hours and the absence of fever or leucocytosis supported this diagnosis. It is acknowledged that aspiration pneumonitis in this context remains a clinical diagnosis, and that sterile endotracheal cultures at 48 hours, while supportive, cannot definitively exclude early aspiration-associated infection, particularly within the first 48 hours of the ictal episode. The empirical initiation of piperacillin-tazobactam reflects this acknowledged diagnostic uncertainty within the initial resuscitation phase. Ventilator-associated lung injury was excluded: CT imaging was performed on hospital day 2, prior to any sustained period of mechanical ventilatory support.

Clinical reasoning

The bronchoscopic findings established a minimum Myer-Cotton Grade III stenosis. The Myer-Cotton system stratifies subglottic and tracheal stenosis by percentage of luminal obstruction: Grade I (0-50%), Grade II (51-70%), Grade III (71-99%), and Grade IV (no detectable lumen) [8]. Inability to advance the bronchoscope was consistent with severe Grade III-IV stenosis; formal quantitative luminal assessment was not feasible in this emergency resuscitation setting. The CT finding of circumferential wall thickening extending 2.3 cm corroborated bronchoscopic severity: stenotic segments exceeding 1 cm in length are not amenable to bronchoscopic dilatation alone and require surgical tracheal resection with end-to-end anastomosis [8].

Airway management followed an escalating stepwise pathway consistent with Difficult Airway Society (DAS) guidelines [6]: Plan A (direct laryngoscopy, failed across three sequential intubation attempts); Plan B (supraglottic LMA bridge); and Plan D (emergency FONA). Cricothyrotomy was not performed. The stenosis was located immediately below the subglottis, directly distal to the cricothyroid membrane; a cricothyrotomy would have placed the rescue airway at or proximal to the obstruction, failing to bypass it entirely. Surgical tracheostomy placed in the mid-trachea, distal to the stenotic segment, was the anatomically appropriate rescue strategy in this specific clinical context, where the stenotic lesion was situated immediately distal to the cricothyroid membrane [6,7]. This case illustrates that successful CICO management demands anatomical contextualisation, not merely algorithmic adherence.

**Figure 2:**
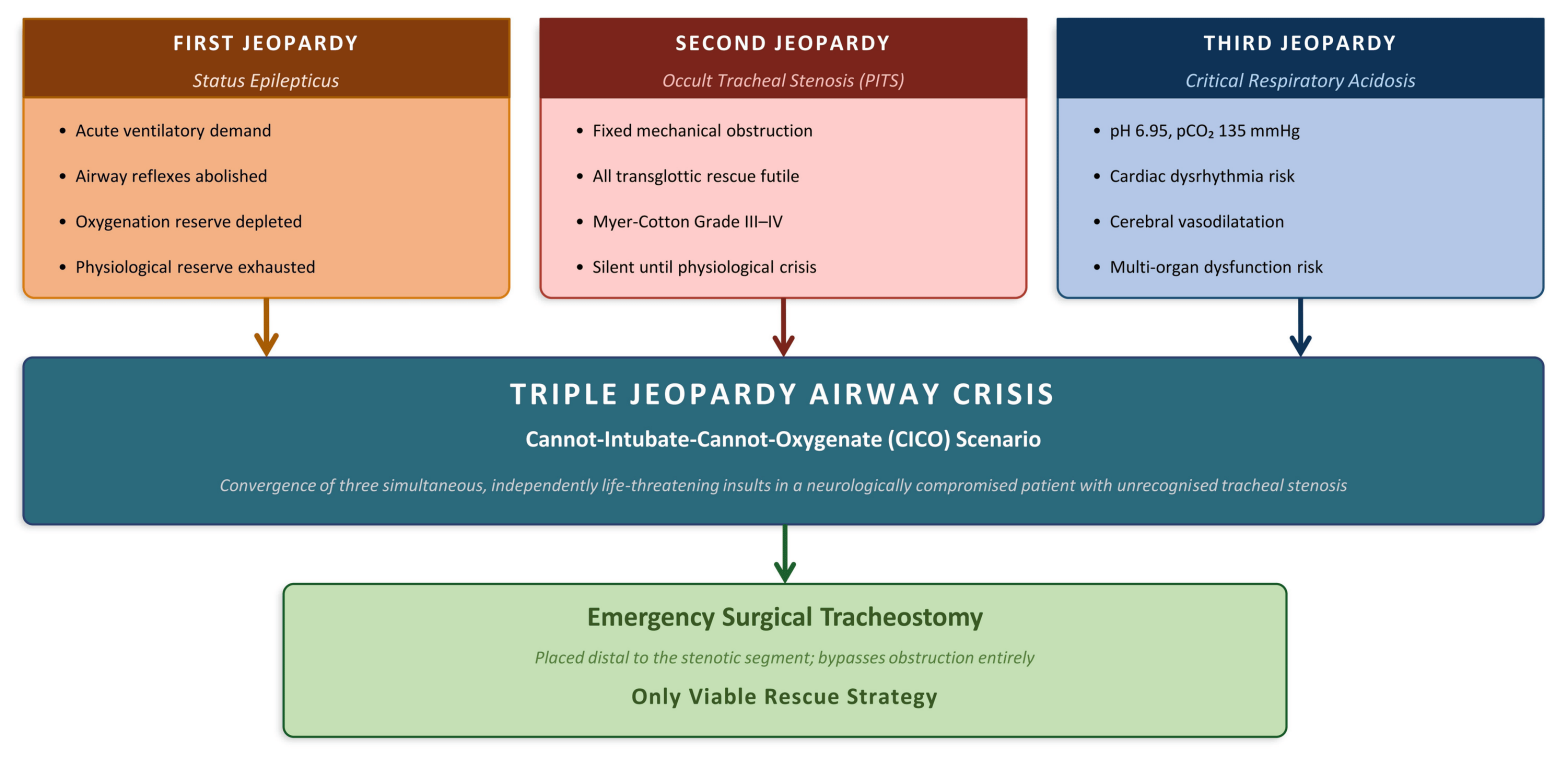
The triple jeopardy airway crisis framework. Three simultaneously life-threatening insults converge to produce a CICO scenario: active status epilepticus abolishes airway protective reflexes and imposes acute ventilatory demand (first jeopardy); occult PITS renders all transglottic rescue futile (second jeopardy); and critical respiratory acidosis independently risks cardiac dysrhythmia and cerebral vasodilatation (third jeopardy). Emergency surgical tracheostomy placed distal to the stenotic segment was the anatomically appropriate rescue strategy in this case. CICO, cannot intubate, cannot oxygenate; pCO_2_, partial pressure of carbon dioxide; PITS, post-intubation tracheal stenosis. The figure was created using Microsoft PowerPoint (Microsoft Corporation, Redmond, WA, USA).

The convergence of three simultaneously life-threatening insults defines the triple jeopardy airway crisis introduced in this report (Figure 2). Active SE imposed acute ventilatory demand and abolished airway protective reflexes (first jeopardy). Occult subglottic tracheal stenosis rendered all transglottic rescue futile (second jeopardy). The resulting critical respiratory acidosis, pH 6.95 and pCO_2_ 135 mmHg, independently risked cardiac dysrhythmia, cerebral vasodilatation, and multi-organ dysfunction (third jeopardy) [3,5]. The silent natural history of PITS explains why this crisis occurred without prior clinical warning: in a post-TBI patient with reduced physical activity, the symptomatic threshold for tracheal stenosis was never reached until the high ventilatory demand of SE eliminated all remaining reserve precipitously [3]. 

Treatment pathway

Phase 1: Emergency Airway Rescue and Resuscitation (Hospital Days 1-2).

Following failure of three sequential endotracheal intubation attempts, a classic LMA was inserted as a bridge while emergency bronchoscopy was performed. Emergency surgical tracheostomy under local anaesthesia bypassed the stenotic segment entirely and restored effective ventilation. For seizure termination, intravenous (IV) lorazepam was administered as first-line benzodiazepine therapy, consistent with current evidence-based guidelines for convulsive SE [12]. IV levetiracetam was administered as second-line loading, in accordance with the Established Status Epilepticus Treatment Trial (ESETT), which demonstrated levetiracetam to be an effective second-tier agent for benzodiazepine-refractory SE [13]. IV midazolam and atracurium were used for sedoanalgesia and neuromuscular blockade. Empirical IV piperacillin-tazobactam was initiated, given illness severity and the clinical inability to exclude aspiration-associated infection within 48 hours. Full medication details are provided in Table 2.

Phase 2: Pulmonary Stabilisation (Hospital Days 3-5).

The patient was maintained on volume-controlled assist-control (V-A/C) mechanical ventilation with serial ABG-guided titration. A transient episode of relative over-ventilation identified on hospital day 4 (pH 7.53, pCO2 26 mmHg) was corrected by reducing the set respiratory rate; ABG normalisation was confirmed on hospital day 5.

Phase 3: Weaning and Recovery (Hospital Days 6-10).

Ventilatory support was progressively reduced from V-A/C to continuous positive airway pressure (CPAP), then to a T-piece spontaneous breathing trial, which the patient tolerated with sustained SpO_2_ above 98% [14]. Chest and limb physiotherapy was initiated throughout this phase. A cutaneous fungal infection identified on the abdominal wall was treated with topical antifungal therapy.

Phase 4: Multidisciplinary Review and Discharge (Hospital Day 11).

The ear, nose, and throat (ENT) and cardiothoracic and vascular surgery (CTVS) teams reviewed the patient jointly. Definitive tracheal resection with end-to-end anastomosis was confirmed as the planned outpatient intervention, to be undertaken following physiological stabilisation and pre-surgical anatomical planning.

Outcomes

The patient was successfully weaned from controlled mechanical ventilation and tolerated a spontaneous breathing trial without recurrent respiratory distress or haemodynamic instability. No recurrence of seizure activity was documented after initiation of definitive airway control and antiepileptic therapy. Endotracheal secretion cultures remained sterile, and pulmonary infiltrates resolved without antibiotic escalation, confirming aspiration pneumonitis as the primary pulmonary diagnosis. Peripheral eosinophilia identified on hospital day 8 (eosinophils 7.5%), temporally associated with piperacillin-tazobactam administration, was flagged for outpatient haematological review following antibiotic cessation [15].

The patient was discharged in stable condition on hospital day 11, breathing spontaneously through the tracheostomy, with structured follow-up under the ENT and CTVS teams. Definitive tracheal resection with end-to-end anastomosis, the gold-standard intervention for Grade III-IV PITS and associated with durable long-term patency superior to endoscopic stenting, was planned for the outpatient phase [8,16]. No post-discharge outcome data were available to the authors at the time of manuscript preparation; the absence of long-term follow-up data on tracheal reconstruction is acknowledged as a limitation of this acute inpatient case report.

## Conclusions

PITS may remain clinically silent in post-traumatic brain injury paediatric patients until a superimposed physiological stressor eliminates residual ventilatory reserve and precipitates an acute airway crisis. Any child with a history of prolonged endotracheal intubation should be regarded as being at risk for occult tracheal stenosis during any subsequent acute airway emergency; this risk is not uniform and is modulated by factors including duration of intubation, cuff inflation pressures, the presence of tracheal mucosal injury, and individual patient characteristics. When occult PITS converges with active status epilepticus and critical respiratory acidosis, the resulting triple jeopardy airway crisis represents the highest-risk clinical pattern in which standard CICO algorithms are insufficient without anatomical contextualisation. This framework is proposed as a descriptive construct from a single case and does not represent a validated clinical entity.

When subglottic obstruction is present or suspected, cricothyrotomy places the rescue airway proximal to the obstruction and will fail; in this anatomical context, only surgical tracheostomy placed distal to the stenotic segment could bypass the obstruction entirely. Emergency bedside flexible bronchoscopy is indispensable in unexplained CICO scenarios, establishing the site and severity of obstruction in real time, directing the choice and level of the surgical airway, and informing subsequent multidisciplinary reconstruction planning, all within the resuscitation window.
